# Supplementary material for: MR-link-2: pleiotropy robust cis Mendelian randomization validated in three independent reference datasets of causality
Source: Nat Commun. 2025 Jul 3;16:6112. doi: 10.1038/s41467-025-60868-1 (PMC12229666; doi:10.1038/s41467-025-60868-1)
Supplement: Supplementary file 1 — Supplementary information [file 41467_2025_60868_MOESM1_ESM.pdf]

Supplementary Information for MR-link-2: pleiotropy robust *cis* Mendelian randomization validated in three independent reference datasets of causality

Supplementary Note

The assumptions underlying MR and analogies to a randomized control trial (RCT)

Mendelian randomization (MR) is a statistical technique that can identify causal relationships from observational data. MR identifies a causal relationship in a process that can be related to a randomized control trial (RCT), with violations of the assumptions paralleling caveats in the RCT design[1]. At conception, all humans randomly receive alleles from their parents. Some of these alleles have effects on human traits which allows us to group individuals into 'genetic treatment' groups based on the genetic variants they carry. MR identifies a causal relationship if the genetic treatment of the risk factor of interest is proportional to the genetics of the outcome trait under investigation. After all, if A is causal to B, all the genetics of trait A should also be visible in the genetics of trait B.

MR is valid under three main assumptions that can also be seen as incorrect applications of RCTs:

i) The relevance assumption states that the variants selected for the genetic treatment, also known as instrumental variables, should be relevant to the risk factor under investigation (**Figure 1a**). In an RCT, this assumption would be violated when the treatment group receives a treatment that has no effect on the risk factor of interest. In MR, genetic variants are usually selected if strongly associated with the risk factor to avoid violating this assumption.

ii) The independence assumption states that the genetic variants under investigation should be independent from confounding with the outcome (**Figure 1a**). In an RCT, this assumption would be violated when there is incorrect randomization. For instance, putting all the smokers in the control group will likely influence trial outcome. In MR this is difficult to test for, as unobserved population stratification can be a source of this violation[2]. Recent evidence suggests that the independence assumption could be violated in the presence of population stratification, assortative mating and dynastic effects. This assumption is difficult to explore in the absence of family data.

iii) The exclusion restriction (also known as horizontal pleiotropy) states that the genetic variant should only affect the outcome through paths that are completely mediated by the risk factor (**Figure 1a**). In an RCT, this assumption would be violated when the treatment contains some form of contamination that also affects the outcome. In this case it is impossible to discern if the treatment has an effect or if it is due to the contamination. In MR, it is difficult to test for the exclusion restriction as the genetic variants selected as instrumental variables that are used for the 'genetic treatment' can have unknown effects that are also causal to the outcome.

Le Chateliers principle and MR on metabolism

Le Chateliers principle states that an increase in concentrations of a substrate also increases the concentrations of a product in a system that is in equilibrium. When applying Mendelian randomization (MR) on metabolites that are in a chemical reaction with one another, we expect that the causal estimate will be positive: an increase in the substrate (exposure in MR) will also increase the product (outcome in MR). One important caveat is that this positive effect is not what is initially expected when the genetic variants that are used for MR causally affect the enzyme that catalyzes a reaction. Interestingly, such a variant will have an effect on both the substrate concentrations and the product concentrations. If the genetic variant reduces the conversion efficiency of the enzyme, this will lead to an increase in the substrate concentrations and a decrease in the product concentrations as less substrate is converted. The MR effect should then seemingly be negative. Interestingly, this case is form of a violation of the exclusion restriction (no horizontal pleiotropy assumption) in an MR framework. The genetic variant that is used will have an effect on the outcome (the concentration of the product in a reaction) that is not directly mediated by the exposure (substrate). On top of this, this also violates *Le Chateliers* principle, as the equilibrium of the reaction is changed between carriers of the variant compared to non-carriers. So, even though a positive MR estimate is seemingly not expected when a genetic variant affects the enzyme catalyzing a reaction, due to a violation of the exclusion restriction as well as *Le Chateliers* principle not holding, the MR estimate should still remain positive in these cases.

eQTLGen cohort information

The interim freeze of eQTLGen genome-wide meta-analysis consisted of 14859 samples from 19 individual datasets from following cohorts: BIOS[3], EstBB[4], Fehrmann Study[5,6], GTEx v8[7–9], INTERVAL[10], KORA[11–14], Young Finns Study[15–17], InCHIANTI[18], Rotterdam Study[19] and BSGS[20,21].

The details for the majority of eQTLGen cohorts used in the preliminary meta-analysis freeze are detailed in the Võsa & Claringbould et al., 2021, and corresponding original publications. Below, we provide the details of the additional INTERVAL cohort, which was not part of Võsa & Claringbould et al., 2021[10,22].

The eQTLGen phase II research activities involving Estonian Biobank participant data (two EstBB cohorts) have been carried out under the ethical approval nr. 1.1-12/655 and its extension 1.1-12/490 by the Estonian Committee on Bioethics and Human Research (Estonian Ministry of Social Affairs), using data according to release application number S54 from the Estonian Biobank. The **Estonian Biobank research team includes** Andres Metspalu, Lili Milani, Tõnu Esko, Reedik Mägi, Mait Metspalu, Mari Nelis, and Georgi Hudjashov. Data analysis was carried out in part in the High-Performance Computing Center of University of Tartu[23].

INTERVAL

The INTERVAL study is a prospective cohort study of approximately 50,000 participants nested within a randomized trial of varying blood donation intervals[24,25]. Between 2012 and 2014, blood donors aged 18 years and older were recruited at 25 centers of England's National Health Service Blood and Transplant (NHSBT). Participants were generally in good health as blood donation criteria exclude individuals with a history of major diseases (e.g. myocardial infarction, stroke, cancer, HIV, and hepatitis B or C) and who have had a recent illness or infection. Participants completed an online questionnaire comprising questions on demographic characteristics (e.g. age, sex, ethnicity), lifestyle (e.g. alcohol and tobacco

consumption), selfreported height and weight, diet and use of medications. All participants gave informed consent before joining the study and the National Research Ethics Service approved this study (11/EE/0538).

Blood collection. Blood samples were collected from all INTERVAL participants at baseline and also from ~60% of participants approximately 24 months after baseline. For a subset of ~5,000 participants at the 24-month time point, an aliquot of 3 ml of whole blood was collected in Tempus Blood RNA Tubes (ThermoFisher Scientific), following the manufacturer's instructions, and then transferred at ambient temperature to the UK Biocentre (Stockport, UK). Samples were stored at -80°C until use.

RNA extraction. RNA extraction was performed by QIAGEN Genomic Services using QIAGEN's proprietary silica technology. The quality control of the extracted RNA was performed by spectrophotometric measurement on an Infinite 200 Microplate Reader (Tecan). RNA Integrity Number (RIN) values were determined using a TapeStation 4200 system (Agilent), following the manufacturer's protocol. Samples with a concentration

Automated RNA-seq library preparation. Samples were quantified with a QuantiFluor RNA System (Promega) using a Mosquito LV liquid handling platform (SPT Labtech), Bravo automation system (Agilent) and FLUOstar Omega plate reader (BMG Labtech), and then cherry-picked to 200 ng in 50 µl (= 4 ng/µl) using a liquid handling platform (Tecan Freedom EVO). Next, mRNA was isolated using a NEBNext Poly(A) mRNA Magnetic Isolation Module (NEB) and then re-suspended in nuclease-free water. Globin depletion was performed using a KAPA RiboErase Globin Kit (Roche). RNA library preparation was done using a NEBNext Ultra II DNA Library Prep Kit for Illumina (NEB) on a Bravo NGS workstation automation system (Agilent). PCR was performed using a KapaHiFi HotStart ReadyMix (Roche) and unique dual-indexed tag barcodes on a Bravo NGS workstation automation system (Agilent). We applied the following PCR programme: 45 sec at 98°C, 14 cycles of 15 sec at 98°C, 30 sec at 65°C and 30 sec at 72°C, followed by 60 sec at 72°C. Using a Zephyr liquid handling platform (PerkinElmer), PCR products were purified using AMPure XP SPRI beads (Agencourt) at a 0.8:1 bead:sample ratio and then eluted in 20 µl of Elution Buffer (QIAGEN). RNA-seq libraries were quantified with an AccuClear Ultra High Sensitivity dsDNA Quantitation Kit (Biotium) using a Mosquito LV liquid handling platform (SPT Labtech), Bravo automation system (Agilent) and FLUOstar Omega plate reader (BMG Labtech). Then, libraries were pooled up to 95-plex in equimolar amounts on a Biomek NX-8 liquid handling

platform (Beckman Coulter), quantified using a High Sensitivity DNA Kit on a 2100 Bioanalyzer (Agilent), and then normalized to 2.8 nM prior to sequencing.

RNA sequencing and data pre-processing. Samples were sequenced using 75 bp paired-end sequencing reads (reverse stranded) on a NovaSeq 6000 system (S4 flow cell, Xp workflow; Illumina). The sequencing data were de-plexed into separate CRAM files for each library in a lane. Adapters that had been hard-clipped prior to alignment were reinserted as soft-clipped post alignment, and duplicated fragments were marked in the CRAM files. The data pre-processing, including sequence QC, and STAR and alignments was performed with the Nextflow pipeline publicly available at https://github.com/wtsi-hgi/nextflowpipelines/blob/rna_seq_interval_5591/pipelines/rna_seq.nf, including the specific aligner parameters. We assessed the sequence data quality using FastQC v0.11.8. Samples mismatched between RNA-seq and genotyping data within the cohort were identified using QTLtools MBV v1.2[26]. Reads were aligned to the GRCh38 human reference genome (Ensembl GTF annotation v99) using STAR v2.7.3a[27]. The STAR index was built against GRCh38 Ensembl GTF v99 using the option -sjdbOverhang 75. STAR was run in a two-pass setup with standard ENCODE options to increase mapping accuracy: (i) a first alignment step of all samples was used to discover novel splice junctions; (ii) splice junctions of all samples from the first step were collected and merged into a single list; (iii) a second step realigned all samples using the merged splice junctions list as input. We used featureCounts v2.0.0[28] to obtain a count matrix.

Gene expression quantification. The raw gene-level count data contained N=60,676 genes across N=4,778 individuals with 2.03–95.55 million uniquely mapped reads (median: ~24 million). Sequencing was performed across 15 batches.

Quality control of gene expression data. We filtered samples of poor quality by removing samples with a read depth below 10 million uniquely mapped reads. A relatedness matrix was obtained using the PLINK v1.9[29] -make-rel 'square' command on pruned genotype data, and a cut-off threshold of 0.1 was used to define related individuals. For each pair of related individuals, one individual was arbitrarily removed. After filtering, the gene expression dataset included 4,731 individuals. We retained 60,580 genes located on autosomal and sex chromosomes. Then, the raw expression matrix was automatically processed with eQTLGen pipelines.

Genotyping data. In brief, DNA extracted from buffy coat samples collected from INTERVAL participants at the study baseline was used to assay approximately 830,000 variants on the Affymetrix Axiom UK Biobank genotyping array[30]. Genotyping and sample QC were performed as previously described[30]. Next, genotype data was automatically quality controlled, pre-phased and imputed by eQTLGen pipelines.

Data availability

The INTERVAL study data used in this paper are available to bona fide researchers from ceudataaccess@medschl.cam.ac.uk. The data access policy for the data is available at http://www.donorhealth-btru.nihr.ac.uk/project/bioresource. The generated RNA-sequencing data have been deposited at the European Genome-phenome Archive (EGA) under the accession number EGAD00001008015.

Acknowledgements

Participants in the INTERVAL randomized controlled trial were recruited with the active collaboration of NHS Blood and Transplant England (https://www.nhsbt.nhs.uk/), which has supported field work and other elements of the trial. DNA extraction and genotyping were co funded by the National Institute for Health and Care Research (NIHR), the NIHR BioResource (https://bioresource.nihr.ac.uk/) and the NIHR Cambridge Biomedical Research Centre (BRC1215-20014) [*]. RNA-seq was funded as part of an alliance between the University of Cambridge and the AstraZeneca Centre for Genomics Research, and by the NIHR Cambridge Biomedical Research Centre (BRC-1215-20014) [*]. The academic coordinating center for INTERVAL was supported by core funding from the NIHR Blood and Transplant Research Unit (BTRU) in Donor Health and Genomics (NIHR BTRU-2014-10024); NIHR BTRU in Donor Health and Behaviour (NIHR203337); UK Medical Research Council (MR/L003120/1); British Heart Foundation (SP/09/002; RG/13/13/30194; RG/18/13/33946); and NIHR Cambridge BRC (BRC-1215-20014; NIHR203312) [*]. A complete list of the investigators and contributors to the INTERVAL trial is provided in Di Angelantonio et al. [24]The academic coordinating center would like to thank blood donor center staff and blood donors for participating in the INTERVAL trial. This work was supported by Health Data Research UK, which is funded by the UK Medical Research Council, Engineering and Physical Sciences Research Council, Economic and Social Research Council, Department of Health and Social Care (England), Chief Scientist Office of the Scottish Government Health and Social Care Directorates, Health and Social Care Research and Development Division (Welsh Government), Public Health Agency (Northern Ireland), British Heart Foundation and

Wellcome. *The views expressed are those of the authors and not necessarily those of the NIHR or the Department of Health and Social Care. The Wellcome Sanger Institute is supported by core funding from the Wellcome Trust (206194 and 220540/Z/20/A). We thank the Wellcome Sanger Institute's Scientific Operations team for their contribution to sequencing data generation. For the purpose of Open Access, the authors have applied a CC BY public copyright licence to any Author Accepted Manuscript version arising from this submission. This work was supported by the Cambridge Service for Data Driven Discovery (CSD3) operated by the University of Cambridge Research Computing Service (https://www.csd3.cam.ac.uk/), provided by Dell EMC and Intel using Tier-2 funding from the Engineering and Physical Sciences Research Council (capital grant EP/P020259/1), and DiRAC funding from the Science and Technology Facilities Council (https://dirac.ac.uk/).

Funding information

E.P. was funded by the EU/EFPIA Innovative Medicines Initiative Joint Undertaking BigData@Heart grant 116074 and is funded by the NIHR BTRU in Donor Health and Behaviour (NIHR203337) [*]. A.T. is supported by the Wellcome Trust (PhD studentship 222548/Z/21/Z). M.I. is supported by the Munz Chair of Cardiovascular Prediction and Prevention and the NIHR Cambridge Biomedical Research Centre (BRC-1215-20014; NIHR203312) [*]. M.I. is also supported by the UK Economic and Social Research Council (ES/T013192/1). M.I. is a trustee of the Public Health Genomics (PHG) Foundation, a member of the Scientific Advisory Board of Open Targets, and has a research collaboration with AstraZeneca that is unrelated to this study. D.S.P. is an employee and stockholder of AstraZeneca.

Derivation of the MR-link 2 likelihood function

Let traits X and Y represent two human traits, where X has a causal effect on Y, its size denoted by α. Moreover, let $\gamma_i^{(x)}$ and $\gamma_i^{(y)}$ represent the multivariable effects of SNP i on X and Y. The vectorized version of these per SNP effects (representing the effect of all SNPs) are analogously denoted $\boldsymbol{\gamma}^{(x)}, \boldsymbol{\gamma}^{(y)}$, respectively. The underlying statistical model is as follows:

$$
\begin{aligned}
X &= G \cdot \boldsymbol{\gamma}^{(x)} + \epsilon_x \\
Y &= \alpha \cdot X + G \cdot \boldsymbol{\gamma}^{(y)} + \epsilon_y
\end{aligned}
$$

with ϵ_x and ϵ_y normally distributed errors. We also assume that the multivariate effect sizes come from a normal distribution, i.e. $\boldsymbol{\gamma}^{(x)} \sim \mathcal{N}(0, \sigma_x^2)$ and $\boldsymbol{\gamma}^{(y)} \sim \mathcal{N}(0, \sigma_y^2)$. Subsequently, let h_x^2 and h_y^2 denote the direct local heritabilities of X and Y, thus $h_x^2 = \sigma_x^2 \cdot m$ and $h_y^2 = \sigma_y^2 \cdot m$. Let us assume that we have now data available for X in a sample of size n_x and for Y in a sample of n_y. For simplicity we assume that both X and Y and the genotype data for each SNP have zero mean and unit variance (across the samples). This determines the error variances as follows: $\epsilon_x \sim \mathcal{N}(0, 1 - h_x^2)$ and $\epsilon_y \sim \mathcal{N}(0, 1 - \alpha^2 - h_y^2)$. By multiplying both sides by G' and divide the first equation by n_x and the second equation by n_y we have

$$
\begin{aligned}
(G' \cdot X)/n_x &= (G' \cdot G/n_x) \cdot \boldsymbol{\gamma}^{(x)} + (G' \cdot \epsilon_x)/n_x \\
(G' \cdot Y)/n_y &= \alpha \cdot (G' \cdot X)/n_y + (G' \cdot G/n_y) \cdot \boldsymbol{\gamma}^{(y)} + (G' \cdot \epsilon_y)/n_y
\end{aligned}
$$

Let us assume that summary statistics (standardised marginal effect size estimates based on the previously stated sample sizes) are available for all m SNPs at a genomic region for trait X and Y, denoted by $\widehat{\beta}_i^{(x)}$ and $\widehat{\beta}_i^{(y)}$ and the corresponding collection of these values in a vector form is $\widehat{\boldsymbol{\beta}}^{(x)}$ and $\widehat{\boldsymbol{\beta}}^{(y)}$. Furthermore, let C be the local $m \times m$ LD matrix. With these notations, we are in position to reformulate the equations as

$$
\begin{aligned}
\widehat{\boldsymbol{\beta}}^{(x)} &= C \cdot \boldsymbol{\gamma}^{(x)} + (G' \cdot \epsilon_x)/n_x \\
\widehat{\boldsymbol{\beta}}^{(y)} &= \alpha \cdot (G' \cdot X)/n_y + C \cdot \boldsymbol{\gamma}^{(y)} + (G' \cdot \epsilon_y)/n_y
\end{aligned}
$$

Substituting into X the first equation (although estimated in a difference sample (of size n_y)) gives

$$
\begin{aligned}
\widehat{\boldsymbol{\beta}}^{(x)} &= C \cdot \boldsymbol{\gamma}^{(x)} + (G' \cdot \epsilon_x)/n_x \\
\widehat{\boldsymbol{\beta}}^{(y)} &= \alpha \cdot \Big(C \cdot \boldsymbol{\gamma}^{(x)} + (G' \cdot \epsilon_x)/n_y\Big) + C \cdot \boldsymbol{\gamma}^{(y)} + (G' \cdot \epsilon_y)/n_y
\end{aligned}
$$

Denoting $\eta_x := (G' \cdot \epsilon_x)/n_x \sim \mathcal{N}(0, C \cdot (1 - h_x^2)/n_x)$ and $\eta_y := \alpha \cdot (G' \cdot \epsilon_x)/n_y + (G' \cdot \epsilon_y)/n_y \sim \mathcal{N}(0, C \cdot (1 - \alpha^2 \cdot h_x^2 - h_y^2)/n_y)$, we have

$$
\begin{aligned}
\widehat{\boldsymbol{\beta}}^{(x)} &= C \cdot \boldsymbol{\gamma}^{(x)} + \eta_x \\
\widehat{\boldsymbol{\beta}}^{(y)} &= C \cdot (\alpha \cdot \boldsymbol{\gamma}^{(x)} + \boldsymbol{\gamma}^{(y)}) + \eta_y
\end{aligned}
$$

Assuming that the marginal effect estimates come from non-overlapping samples (i.e. their errors are uncorrelated), the likelihood function can be written

$$
\begin{aligned}
L\left(\widehat{\boldsymbol{\beta}}^{(x)}, \widehat{\boldsymbol{\beta}}^{(y)} \middle| \alpha, \boldsymbol{\gamma}^{(x)}, \boldsymbol{\gamma}^{(y)}\right) &= \exp\Big(-\frac{n_y}{2} \cdot (\widehat{\boldsymbol{\beta}}^{(y)} - C(\alpha \cdot \boldsymbol{\gamma}^{(x)} + \boldsymbol{\gamma}^{(y)})' \cdot C^{-1}(\widehat{\boldsymbol{\beta}}^{(y)} - C(\alpha \cdot \boldsymbol{\gamma}^{(x)} + \boldsymbol{\gamma}^{(y)})\Big) \\
&\times \det(C/n_y)^{-1/2} \cdot (2\pi)^{-m/2} \\
&\times \exp\Big(-\frac{n_x}{2} \cdot (\widehat{\boldsymbol{\beta}}^{(x)} - C\boldsymbol{\gamma}^{(x)})' \cdot C^{-1}(\widehat{\boldsymbol{\beta}}^{(x)} - C\boldsymbol{\gamma}^{(x)})\Big) \cdot \det(C/n_x)^{-1/2} \cdot (2\pi)^{-m/2}
\end{aligned}
$$

If we set the following priors for $\boldsymbol{\gamma}^{(x)}, \boldsymbol{\gamma}^{(y)}$, reflecting the InSIDE assumption (covariance being zero):

$$\begin{pmatrix} \boldsymbol{\gamma}^{(x)} \\ \boldsymbol{\gamma}^{(y)} \end{pmatrix} \sim \mathcal{N}\left(\begin{pmatrix} 0 \\ 0 \end{pmatrix}, \begin{pmatrix} \sigma_x^2 \cdot I & 0 \\ 0 & \sigma_y^2 \cdot I \end{pmatrix}\right)$$

We can write

$$\begin{aligned}
L\left(\widehat{\boldsymbol{\beta}}^{(x)}, \widehat{\boldsymbol{\beta}}^{(y)} \,|\alpha, \sigma_x^2, \sigma_y^2\right) &= \int\int \exp\left(-\frac{n_y}{2} \cdot (\widehat{\boldsymbol{\beta}}^{(y)} - C(\alpha \cdot \boldsymbol{\gamma}^{(x)} + \boldsymbol{\gamma}^{(y)})' \cdot C^{-1}(\widehat{\boldsymbol{\beta}}^{(y)} - C(\alpha \cdot \boldsymbol{\gamma}^{(x)} + \boldsymbol{\gamma}^{(y)})\right) \\
&\times \det(C/n_y)^{-1/2} \cdot (2\pi)^{-m/2} \\
&\times \exp\left(-\frac{n_x}{2} \cdot (\widehat{\boldsymbol{\beta}}^{(x)} - C\boldsymbol{\gamma}^{(x)})' \cdot C^{-1}(\widehat{\boldsymbol{\beta}}^{(x)} - C\boldsymbol{\gamma}^{(x)})\right) \cdot \det(C/n_x)^{-1/2} \cdot (2\pi)^{-m/2} \\
&\times \left(\frac{1}{\sqrt{2\pi}\sigma_x}\right)^m \cdot \exp\left(-\frac{1}{2\sigma_x^2} \cdot (\boldsymbol{\gamma}^{(x)})' \cdot \boldsymbol{\gamma}^{(x)}\right) \\
&\times \left(\frac{1}{\sqrt{2\pi}\sigma_y}\right)^m \cdot \exp\left(-\frac{1}{2\sigma_y^2} \cdot (\boldsymbol{\gamma}^{(y)})' \cdot \boldsymbol{\gamma}^{(y)}\right) d\boldsymbol{\gamma}^{(x)} d\boldsymbol{\gamma}^{(y)} \\
&= \int\int \exp\left(-\frac{n_y}{2} \cdot (\widehat{\boldsymbol{\beta}}^{(y)})' C^{-1} \widehat{\boldsymbol{\beta}}^{(y)}\right) \cdot \exp\left(-\frac{n_y}{2} \cdot \alpha^2 \cdot (\boldsymbol{\gamma}^{(x)})' C \boldsymbol{\gamma}^{(x)}\right) \\
&\times \exp\left(-\frac{n_y}{2} \cdot (\boldsymbol{\gamma}^{(y)})' C \boldsymbol{\gamma}^{(y)}\right) \cdot \exp\left(-\frac{n_y}{2} \cdot (-2\alpha) \cdot (\widehat{\boldsymbol{\beta}}^{(y)})' \boldsymbol{\gamma}^{(x)}\right) \\
&\times \exp\left(-\frac{n_y}{2} \cdot (-2) \cdot (\widehat{\boldsymbol{\beta}}^{(y)})' \boldsymbol{\gamma}^{(y)}\right) \\
&\times \exp\left(-\frac{n_y}{2} \cdot (2) \cdot \alpha \cdot (\boldsymbol{\gamma}^{(x)})' \cdot C \cdot \boldsymbol{\gamma}^{(y)}\right) \\
&\times \exp\left(-\frac{n_x}{2} \cdot (\widehat{\boldsymbol{\beta}}^{(x)})' C^{-1} \widehat{\boldsymbol{\beta}}^{(x)}\right) \cdot \exp\left(-\frac{n_x}{2} \cdot (\boldsymbol{\gamma}^{(x)})' C \boldsymbol{\gamma}^{(x)}\right) \\
&\times \exp\left(-\frac{n_x}{2} \cdot (-2) \cdot (\widehat{\boldsymbol{\beta}}^{(x)})' \boldsymbol{\gamma}^{(x)}\right) \\
&\times \exp\left(-\frac{1}{2\sigma_x^2} \cdot (\boldsymbol{\gamma}^{(x)})' \cdot \boldsymbol{\gamma}^{(x)}\right) \cdot \exp\left(-\frac{1}{2\sigma_y^2} \cdot (\boldsymbol{\gamma}^{(y)})' \cdot \boldsymbol{\gamma}^{(y)}\right) \\
&\times (2\pi)^{-2m} \cdot n_y^{m/2} n_x^{m/2} \det(C)^{-1} \cdot \sigma_x^{-m} \cdot \sigma_y^{-m} d\boldsymbol{\gamma}^{(x)} d\boldsymbol{\gamma}^{(y)}
\end{aligned}$$

Next we complete the squares for $\boldsymbol{\gamma}^{(x)}$ and $\boldsymbol{\gamma}^{(y)}$

$$\begin{aligned}
L\left(\widehat{\boldsymbol{\beta}}^{(x)}, \widehat{\boldsymbol{\beta}}^{(y)} \,|\alpha, \sigma_x^2, \sigma_y^2\right) &= \int\int \exp\left(-\frac{1}{2} \cdot (\boldsymbol{\gamma}^{(x)})' \left((\alpha^2 \cdot n_y + n_x) \cdot C + \sigma_x^{-2} \cdot I\right) \boldsymbol{\gamma}^{(x)}\right) \\
&\times \exp\left(-\frac{1}{2} \cdot (\boldsymbol{\gamma}^{(y)})' \left(n_y \cdot C + \sigma_y^{-2} \cdot I\right) \boldsymbol{\gamma}^{(y)}\right) \\
&\times \exp\left(-\frac{1}{2} \cdot (2) \cdot (\boldsymbol{\gamma}^{(x)})' \left(\alpha \cdot n_y \cdot C\right) \boldsymbol{\gamma}^{(y)}\right) \\
&\times \exp\left(-\frac{1}{2} \cdot (-2) \cdot (n_y \cdot \widehat{\boldsymbol{\beta}}^{(y)})' \boldsymbol{\gamma}^{(y)}\right) \\
&\times \exp\left(-\frac{1}{2} \cdot (-2) \cdot (n_x \cdot \widehat{\boldsymbol{\beta}}^{(x)} + \alpha \cdot n_y \cdot \widehat{\boldsymbol{\beta}}^{(y)})' \boldsymbol{\gamma}^{(x)}\right) \\
&\times \exp\left(-\frac{n_x}{2} \cdot (\widehat{\boldsymbol{\beta}}^{(x)})' C^{-1} \widehat{\boldsymbol{\beta}}^{(x)}\right) \cdot \exp\left(-\frac{n_y}{2} \cdot (\widehat{\boldsymbol{\beta}}^{(y)})' C^{-1} \widehat{\boldsymbol{\beta}}^{(y)}\right) \\
&\times (2\pi)^{-2m} \cdot n_y^{m/2} n_x^{m/2} \det(C)^{-1} \cdot \sigma_x^{-m} \cdot \sigma_y^{-m} d\boldsymbol{\gamma}^{(x)} d\boldsymbol{\gamma}^{(y)}
\end{aligned}$$

By introducing the following notations

$$
\begin{aligned}
\Omega_{x,x} &:= \left((\alpha^2 \cdot n_y + n_x) \cdot C + \sigma_x^{-2} \cdot I\right) \\
\Omega_{y,y} &:= \left(n_y \cdot C + \sigma_y^{-2} \cdot I\right) \\
\Omega_{x,y} &:= (\alpha \cdot n_y \cdot C) \\
\boldsymbol{\mu}_x &:= n_x \cdot \widehat{\boldsymbol{\beta}}^{(x)} + \alpha \cdot n_y \cdot \widehat{\boldsymbol{\beta}}^{(y)} \\
\boldsymbol{\mu}_y &:= n_y \cdot \widehat{\boldsymbol{\beta}}^{(y)}
\end{aligned}
$$

and using these notations further shorthand can be used

$$
\begin{aligned}
\Omega : &= \begin{pmatrix} \Omega_{x,x} & \Omega_{x,y} \\ \Omega_{x,y} & \Omega_{y,y} \end{pmatrix} \\
\boldsymbol{\mu} &:= \begin{pmatrix} \boldsymbol{\mu}_x \\ \boldsymbol{\mu}_y \end{pmatrix} \\
\boldsymbol{\gamma} &:= \begin{pmatrix} \boldsymbol{\gamma}^{(x)} \\ \boldsymbol{\gamma}^{(y)} \end{pmatrix}
\end{aligned}
$$

This allows us to turn the likelihood function into

$$
\begin{aligned}
L\left(\widehat{\boldsymbol{\beta}}^{(x)}, \widehat{\boldsymbol{\beta}}^{(y)} \middle| \alpha, \sigma_x^2, \sigma_y^2\right) &= \int\int \exp\left(-\frac{1}{2} \cdot (\boldsymbol{\gamma})' \cdot \Omega \cdot \boldsymbol{\gamma}\right) \cdot \exp\left(-\frac{1}{2} \cdot (-2) \cdot \boldsymbol{\mu}' \cdot \boldsymbol{\gamma}\right) d\boldsymbol{\gamma} \\
&\times \exp\left(-\frac{n_x}{2} \cdot (\widehat{\boldsymbol{\beta}}^{(x)})' C^{-1} \widehat{\boldsymbol{\beta}}^{(x)}\right) \cdot \exp\left(-\frac{n_y}{2} \cdot (\widehat{\boldsymbol{\beta}}^{(y)})' C^{-1} \widehat{\boldsymbol{\beta}}^{(y)}\right) \\
&\times (2\pi)^{-2m} \cdot n_y^{m/2} n_x^{m/2} \det(C)^{-1} \cdot \sigma_x^{-m} \cdot \sigma_y^{-m} \\
&= \int \exp\left(-\frac{1}{2} \cdot (\boldsymbol{\gamma} - \Omega^{-1} \cdot \boldsymbol{\mu})' \cdot \Omega \cdot (\boldsymbol{\gamma} - \Omega^{-1} \cdot \boldsymbol{\mu})\right) d\boldsymbol{\gamma} \\
&\times \exp\left(\frac{1}{2} \boldsymbol{\mu}' \cdot \Omega^{-1} \cdot \boldsymbol{\mu}\right) \\
&\times \exp\left(-\frac{n_x}{2} \cdot (\widehat{\boldsymbol{\beta}}^{(x)})' C^{-1} \widehat{\boldsymbol{\beta}}^{(x)}\right) \cdot \exp\left(-\frac{n_y}{2} \cdot (\widehat{\boldsymbol{\beta}}^{(y)})' C^{-1} \widehat{\boldsymbol{\beta}}^{(y)}\right) \\
&\times (2\pi)^{-2m} \cdot n_y^{m/2} n_x^{m/2} \det(C)^{-1} \cdot \sigma_x^{-m} \cdot \sigma_y^{-m} \\
&= \int \exp\left(-\frac{1}{2} \cdot (\boldsymbol{\gamma} - \Omega^{-1} \cdot \boldsymbol{\mu})' \cdot \Omega \cdot (\boldsymbol{\gamma} - \Omega^{-1} \cdot \boldsymbol{\mu})\right) \cdot (2\pi)^{-m} \det(\Omega)^{1/2} d\boldsymbol{\gamma} \\
&\times (2\pi)^{m} \det(\Omega)^{-1/2} \cdot \exp\left(\frac{1}{2} \boldsymbol{\mu}' \cdot \Omega^{-1} \cdot \boldsymbol{\mu}\right) \\
&\times \exp\left(-\frac{n_x}{2} \cdot (\widehat{\boldsymbol{\beta}}^{(x)})' C^{-1} \widehat{\boldsymbol{\beta}}^{(x)}\right) \cdot \exp\left(-\frac{n_y}{2} \cdot (\widehat{\boldsymbol{\beta}}^{(y)})' C^{-1} \widehat{\boldsymbol{\beta}}^{(y)}\right) \\
&\times (2\pi)^{-2m} \cdot n_y^{m/2} n_x^{m/2} \det(C)^{-1} \cdot \sigma_x^{-m} \cdot \sigma_y^{-m} \\
&= (2\pi)^{m} \det(\Omega)^{-1/2} \cdot \exp\left(\frac{1}{2} \boldsymbol{\mu}' \cdot \Omega^{-1} \cdot \boldsymbol{\mu}\right) \\
&\times \exp\left(-\frac{n_x}{2} \cdot (\widehat{\boldsymbol{\beta}}^{(x)})' C^{-1} \widehat{\boldsymbol{\beta}}^{(x)}\right) \cdot \exp\left(-\frac{n_y}{2} \cdot (\widehat{\boldsymbol{\beta}}^{(y)})' C^{-1} \widehat{\boldsymbol{\beta}}^{(y)}\right) \\
&\times (2\pi)^{-2m} \cdot n_y^{m/2} n_x^{m/2} \det(C)^{-1} \cdot \sigma_x^{-m} \cdot \sigma_y^{-m}
\end{aligned}
$$

Let us replace C with its singular value decomposition $U \cdot \Lambda \cdot U'$ with the ith diagonal element of Λ being λ_i. In addition, let's introduce the notation $\boldsymbol{\nu}_x := U' \cdot \boldsymbol{\mu}_x$ and $\boldsymbol{\nu}_y := U' \cdot \boldsymbol{\mu}_y$ and analogously, $\widehat{\boldsymbol{\delta}}^{(x)} := U' \cdot \widehat{\boldsymbol{\beta}}^{(x)}$ and $\widehat{\boldsymbol{\delta}}^{(y)} := U' \cdot \widehat{\boldsymbol{\beta}}^{(y)}$. This allows the reformulation of $\Omega_{x,x}, \Omega_{y,y}$

and $\Omega_{x,y}$ as follows:

$$\begin{aligned}
\Omega_{x,x} &= U \cdot \left((\alpha^2 \cdot n_y + n_x) \cdot \Lambda + \sigma_x^{-2} \cdot I\right) \cdot U' \\
\Omega_{y,y} &= U \cdot \left(n_y \cdot \Lambda + \sigma_y^{-2} \cdot I\right) U' \\
\Omega_{x,y} &= U \cdot (\alpha \cdot n_y \cdot \Lambda) \, U'
\end{aligned}$$

Using the formula for block matrix determinant, $\det(\Omega) = \det(\Omega_{x,x} - \Omega_{x,y}\Omega_{y,y}^{-1}\Omega_{y,x}) \cdot \det(\Omega_{y,y})$, we have

$$\begin{aligned}
\det(\Omega) &= \det\left((\alpha^2 \cdot n_y + n_x) \cdot \Lambda + \sigma_x^{-2} \cdot I - \alpha^2 \cdot n_y^2 \Lambda \cdot (n_y \cdot \Lambda + \sigma_y^{-2} \cdot I)^{-1} \cdot \Lambda\right) \cdot \det(n_y \cdot \Lambda + \sigma_y^{-2} \cdot I) \\
&= \prod_{i=1}^{m} \left((\alpha^2 \cdot n_y + n_x) \cdot \lambda_i + \sigma_x^{-2} - \frac{\alpha^2 \cdot n_y^2 \cdot \lambda_i^2}{n_y \cdot \lambda_i + \sigma_y^{-2}}\right) \cdot \prod_{i=1}^{m} \left(n_y \cdot \lambda_i + \sigma_y^{-2}\right) \qquad (1)
\end{aligned}$$

This time the formula of the block matrix inverse allows us the calculation of the block elements of the inverse of Ω:

$$\begin{pmatrix} (\Omega^{-1})_{x,x} & (\Omega^{-1})_{x,y} \\ (\Omega^{-1})_{y,x} & (\Omega^{-1})_{y,y} \end{pmatrix} := \begin{pmatrix} \Omega_{x,x} & \Omega_{x,y} \\ \Omega_{x,y} & \Omega_{y,y} \end{pmatrix}^{-1}$$

The first block can be written as

$$\begin{aligned}
(\Omega^{-1})_{x,x} &= \left(\Omega_{x,x} - \Omega_{x,y} \cdot \Omega_{y,y}^{-1} \cdot \Omega_{y,x}\right)^{-1} \\
&= U \cdot \underbrace{\left(\left((\alpha^2 \cdot n_y + n_x) \cdot \Lambda + \sigma_x^{-2} \cdot I\right) - (\alpha \cdot n_y \cdot \Lambda) \cdot \left(n_y \cdot \Lambda + \sigma_y^{-2} \cdot I\right)^{-1} \cdot (\alpha \cdot n_y \cdot \Lambda)\right)^{-1}}_{D^{(x,x)}} \cdot U'
\end{aligned}$$

where $D^{(x,x)}$ is a diagonal matrix with elements

$$D_{i,i}^{(x,x)} = \left(\left((\alpha^2 \cdot n_y + n_x) \cdot \lambda_i + \sigma_x^{-2}\right) - \frac{\alpha^2 \cdot n_y^2 \cdot \lambda_i^2}{n_y \cdot \lambda_i + \sigma_y^{-2}}\right)^{-1} \qquad (2)$$

The second block can be written as

$$\begin{aligned}
(\Omega^{-1})_{x,y} &= -\left(\Omega_{x,x} - \Omega_{x,y} \cdot \Omega_{y,y}^{-1} \cdot \Omega_{y,x}\right)^{-1} \cdot \Omega_{x,y} \cdot \Omega_{y,y}^{-1} \\
&= -U \cdot D^{(x,x)} \cdot (\alpha \cdot n_y \cdot \Lambda) \cdot \left(n_y \cdot \Lambda + \sigma_y^{-2} \cdot I\right)^{-1} \cdot U' \\
&= U \cdot D^{(x,y)} \cdot U'
\end{aligned}$$

where $D^{(x,y)}$ is a diagonal matrix with elements

$$D_{i,i}^{(x,y)} = -D_{i,i}^{(x,x)} \cdot \frac{\alpha \cdot n_y \cdot \lambda_i}{n_y \cdot \lambda_i + \sigma_y^{-2}} \qquad (3)$$

Finally, the last block is of the form

$$\begin{aligned}
(\Omega^{-1})_{y,y} &= \Omega_{y,y}^{-1} + \Omega_{y,y}^{-1} \cdot \Omega_{y,x} \cdot \left(\Omega_{x,x} - \Omega_{x,y} \cdot \Omega_{y,y}^{-1} \cdot \Omega_{y,x}\right)^{-1} \cdot \Omega_{x,y}\Omega_{y,y}^{-1} \\
&= U \cdot \Big(\left(n_y \cdot \Lambda + \sigma_y^{-2} \cdot I\right)^{-1} \\
&+ \left(n_y \cdot \Lambda + \sigma_y^{-2} \cdot I\right)^{-1} \cdot (\alpha \cdot n_y \cdot \Lambda) \cdot D^{(x,x)} \cdot (\alpha \cdot n_y \cdot \Lambda) \cdot \left(n_y \cdot \Lambda + \sigma_y^{-2} \cdot I\right)^{-1}\Big) \cdot U' \\
&= U \cdot D^{(y,y)} \cdot U'
\end{aligned}$$

where $D^{(y,y)}$ is a diagonal matrix with elements

$$D_{i,i}^{(y,y)} = \frac{1}{n_y \cdot \lambda_i + \sigma_y^{-2}} + D_{i,i}^{(x,x)} \cdot \frac{\alpha^2 \cdot n_y^2 \cdot \lambda_i^2}{(n_y \cdot \lambda_i + \sigma_y^{-2})^2} \tag{4}$$

The bilinear product in the likelihood function can be written as

$$\begin{aligned}
\boldsymbol{\mu}' \cdot \Omega^{-1} \cdot \boldsymbol{\mu} &= \boldsymbol{\mu}_x' \cdot (\Omega^{-1})_{x,x} \cdot \boldsymbol{\mu}_x + 2 \cdot \boldsymbol{\mu}_x' \cdot (\Omega^{-1})_{x,y} \cdot \boldsymbol{\mu}_y + \boldsymbol{\mu}_y' \cdot (\Omega^{-1})_{y,y} \cdot \boldsymbol{\mu}_y \\
&= (\boldsymbol{\nu}_x)' \cdot D^{(x,x)} \cdot \boldsymbol{\nu}_x + 2 \cdot (\boldsymbol{\nu}_x)' \cdot D^{(x,y)} \cdot \boldsymbol{\nu}_y + (\boldsymbol{\nu}_y)' \cdot D^{(y,y)} \cdot \boldsymbol{\nu}_y \\
&= \sum_{i=1}^{m} (\boldsymbol{\nu}_x)_i^2 \cdot D_{i,i}^{(x,x)} + 2 \cdot \sum_{i=1}^{m} (\boldsymbol{\nu}_x)_i \cdot (\boldsymbol{\nu}_y)_i \cdot D_{i,i}^{(x,y)} + \sum_{i=1}^{m} (\boldsymbol{\nu}_y)_i \cdot D_{i,i}^{(y,y)}
\end{aligned}$$

Analogously, we have

$$\begin{aligned}
(\widehat{\boldsymbol{\beta}}^{(x)})' C^{-1} \widehat{\boldsymbol{\beta}}^{(x)} &= (\widehat{\boldsymbol{\delta}}^{(x)})' \cdot \Lambda^{-1} \cdot \widehat{\boldsymbol{\delta}}^{(x)} = \sum_{i=1}^{m} (\widehat{\boldsymbol{\delta}}_i^{(x)})^2 / \lambda_i \\
(\widehat{\boldsymbol{\beta}}^{(y)})' C^{-1} \widehat{\boldsymbol{\beta}}^{(y)} &= (\widehat{\boldsymbol{\delta}}^{(y)})' \cdot \Lambda^{-1} \cdot \widehat{\boldsymbol{\delta}}^{(y)} = \sum_{i=1}^{m} (\widehat{\boldsymbol{\delta}}_i^{(y)})^2 / \lambda_i
\end{aligned}$$

This puts us in position to simplify the log-likelihood function to

$$\begin{aligned}
l\left(\widehat{\boldsymbol{\beta}}^{(x)}, \widehat{\boldsymbol{\beta}}^{(y)}\right) &= l\left(\widehat{\boldsymbol{\delta}}^{(x)}, \widehat{\boldsymbol{\delta}}^{(y)}\right) \\
&= -m \cdot \log(2\pi) - \frac{1}{2} \cdot \sum_{i=1}^{m} \log\left((\alpha^2 \cdot n_y + n_x) \cdot \lambda_i + \sigma_x^{-2} - \frac{\alpha^2 \cdot n_y^2 \cdot \lambda_i^2}{n_y \cdot \lambda_i + \sigma_y^{-2}}\right) \\
&- \frac{1}{2} \cdot \sum_{i=1}^{m} \log\left(n_y \cdot \lambda_i + \sigma_y^{-2}\right) \\
&+ \frac{1}{2}\left(\sum_{i=1}^{m} (\widehat{\boldsymbol{\delta}}^{(x)})_i^2 \cdot D_{i,i}^{(x,x)} + 2 \cdot \sum_{i=1}^{m} \widehat{\boldsymbol{\delta}}_i^{(x)} \cdot \widehat{\boldsymbol{\delta}}_i^{(y)} \cdot D_{i,i}^{(x,y)} + \sum_{i=1}^{m} \widehat{\boldsymbol{\delta}}_i^{(y)} \cdot D_{i,i}^{(y,y)}\right) \\
&- \frac{n_x}{2} \cdot (\sum_{i=1}^{m} (\widehat{\boldsymbol{\delta}}_i^{(x)})^2 / \lambda_i) - \frac{n_y}{2} \cdot (\sum_{i=1}^{m} (\widehat{\boldsymbol{\delta}}_i^{(y)})^2 / \lambda_i) \\
&+ \frac{m}{2} \cdot (\log(n_x) + \log(n_y)) - \sum_{i=1}^{m} \log(\lambda_i) - m \cdot (\log(\sigma_x) + \log(\sigma_y))
\end{aligned}$$

Implementation details

We have implemented the MR-link-2 likelihood function. To ensure numerical stability, we have made two purely numerical transformations in the likelihood function. First we $t_X = \sigma_X^{-2}$ and $t_Y = \sigma_Y^{-2}$. This will reduce numerical instability when either σ_X or σ_Y are very close to zero.

The likelihood function of MR-link-2 that is optimized is then:

$$
\begin{aligned}
l\left(\widehat{\boldsymbol{\beta}}^{(x)}, \widehat{\boldsymbol{\beta}}^{(y)}\right) &= l\left(\widehat{\boldsymbol{\delta}}^{(x)}, \widehat{\boldsymbol{\delta}}^{(y)}\right) \\
&= -m \cdot \log(2\pi) - \frac{1}{2} \cdot \sum_{i=1}^{m} \log\left((\alpha^2 \cdot n_y + n_x) \cdot \lambda_i + t_X - \frac{\alpha^2 \cdot n_y^2 \cdot \lambda_i^2}{n_y \cdot \lambda_i + t_Y}\right) \\
&- \frac{1}{2} \cdot \sum_{i=1}^{m} \log\left(n_y \cdot \lambda_i + \sigma_y^{-2}\right) \\
&+ \frac{1}{2}\left(\sum_{i=1}^{m} (\widehat{\boldsymbol{\delta}}^{(x)})_i^2 \cdot D_{i,i}^{(x,x)} + 2 \cdot \sum_{i=1}^{m} \widehat{\boldsymbol{\delta}}_i^{(x)} \cdot \widehat{\boldsymbol{\delta}}_i^{(y)} \cdot D_{i,i}^{(x,y)} + \sum_{i=1}^{m} \widehat{\boldsymbol{\delta}}_i^{(y)} \cdot D_{i,i}^{(y,y)}\right) \\
&- \frac{n_x}{2} \cdot \left(\sum_{i=1}^{m} (\widehat{\boldsymbol{\delta}}_i^{(x)})^2 / \lambda_i\right) - \frac{n_y}{2} \cdot \left(\sum_{i=1}^{m} (\widehat{\boldsymbol{\delta}}_i^{(y)})^2 / \lambda_i\right) \\
&+ \frac{m}{2} \cdot (\log(n_x) + \log(n_y)) - \sum_{i=1}^{m} \log(\lambda_i) + \frac{m}{2} \cdot (\log(t_X) + \log(t_Y))
\end{aligned}
$$

To further reduce numerical instability of the likelihood function, we make use of the exponent logarithm trick to reduce numerical instability: Consider a value x, and then we take the exponent and the logarithm of x, this is equal to $x = exp(log(x))$. If x is defined as a multiplication, $x = a * b$, this allows us to rewrite as $x = exp(log(a) + log(b))$, as $log(a * b) = log(a) + log(b)$. Similarly, if x represents a division: $x = a/b$, this can be represented by $log(a/b) = log(a) - log(b)$

We also perform simplifications when $\alpha = 0$. A copy of the python function is found on the next page

```python
def mr_link2_loglik_reference_v2(th: np.ndarray, lam: np.ndarray,
                                 c_x: np.ndarray, c_y: np.ndarray,
                                 n_x: float, n_y: float) -> float:
    """
    The MR-link2 log likelihood function. This function calculates -1 * likelihood of three parameters:
    alpha, sigma_x and sigma_y.
    Designed to be used in optimization algorithms like those in scipy.minimize

    :param th:
        List or numpy array of floats with the parameters to optimize first is alpha, second the
        1 /  exposure heritability (per variant)  and third the 1/ outcome heritability (per variant).
    :param lam:
        np.ndarray of selected eigenvalues of the cX and cY parameters.
    :param c_x:
        The dot product of the selected eigenvectors and summary statistics vector of the exposure
    :param c_y:
        The dot product of the selected eigenvectors and summary statistics vector of the outcome
    :param n_x:
        The number of individuals in the exposure dataset
    :param n_y:
        The number of individuals in the outcome dataset
    :return:
        a single float that contains the likelihood of the parameters theta.
    """

    n_x = float(n_x)
    n_y = float(n_y)
    a = th[0]
    tX = abs(th[1])
    tY = abs(th[2])

    Dyy = (1. / (n_y * lam + tY))

    if a != 0.0:
        Dxx = 1. / (np.exp(np.log(a ** 2 * n_y + n_x) + np.log(lam)) + tX -
                    np.exp(np.log(a ** 2 * n_y ** 2 * (lam ** 2)) - np.log(n_y * lam + tY)))
        Dxy = -Dxx * a * np.exp(np.log((n_y * lam)) - np.log(n_y * lam + tY))
        Dyy = Dyy + np.exp(np.log(Dxx * (a ** 2 * n_y ** 2 * lam ** 2)) - (2 * np.log(n_y * lam + tY)))
        asq_ny_sq_lam_sq_div_ny_lam_ty = np.exp(np.log(a ** 2 * n_y ** 2 * (lam ** 2)) - np.log(n_y * lam + tY))
    else:
        Dxx = 1. / (np.exp(np.log(n_x) + np.log(lam)) + tX)
        Dxy = -Dxx * a * np.exp(np.log((n_y * lam)) - np.log(n_y * lam + tY))
        Dyy = Dyy
        asq_ny_sq_lam_sq_div_ny_lam_ty = 0.0 * lam

    dX = n_x * c_x + a * n_y * c_y
    dY = n_y * c_y
    m = len(c_x)

    loglik = -m * np.log(2 * np.pi) + \
             -(1 / 2) * sum(np.log((a ** 2 * n_y + n_x) * lam + tX - asq_ny_sq_lam_sq_div_ny_lam_ty)) + \
             -(1 / 2) * sum(np.log(n_y * lam + tY)) + \
             +(1 / 2) * (sum(dX ** 2 * Dxx) + 2 * sum(dX * dY * Dxy) + sum(dY ** 2 * Dyy)) + \
             -(n_x / 2) * sum((c_x ** 2) / lam) + \
             -(n_y / 2) * sum((c_y ** 2) / lam) + \
             +(m / 2) * (np.log(n_x) + np.log(n_y)) - sum(np.log(lam)) + (m / 2) * (np.log(tX) + np.log(tY))

    return -loglik
```

Rescaling genetic effects to standardized effects

Let X and G represent a complex trait and a genetic variant respectively. Let $\boldsymbol{x}, \boldsymbol{g}$ denote their realisations in a sample of n individuals. When fitting the following linear model

$$X = G \cdot \beta + \epsilon \quad \text{with} \quad \epsilon \sim \mathcal{N}\left(0, \sigma^2\right)$$

to these data we obtain a parameter estimate $\widehat{\beta}$ for the slope, which can be written as

$$\widehat{\beta} = \frac{(\boldsymbol{x} - \bar{x})' \cdot (\boldsymbol{g} - \bar{g})}{(\boldsymbol{g} - \bar{g})' \cdot (\boldsymbol{g} - \bar{g})} = \frac{\operatorname{Cov}(\boldsymbol{x}, \boldsymbol{g})}{\operatorname{Var}(\boldsymbol{g})}$$

where $\bar{x}$ refers to the sample mean. And the variance of the estimate is

$$\operatorname{Var}(\widehat{\beta}) = \sigma^2 \cdot \left((\boldsymbol{g} - \bar{g})' \cdot (\boldsymbol{g} - \bar{g})\right)^{-1} = \frac{\sigma^2}{(n-1) \cdot \operatorname{Var}(\boldsymbol{g})}$$

The residual variance can be expressed by equating the variance of the two sides of the model

$$\operatorname{Var}(\boldsymbol{x}) = \widehat{\beta}^2 \cdot \operatorname{Var}(\boldsymbol{g}) + \sigma^2$$

The squared Z-statistic can be written as

$$\begin{aligned} Z^2 &= \frac{\widehat{\beta}^2}{\operatorname{Var}(\widehat{\beta})} = \widehat{\beta}^2 \cdot \frac{(n-1) \cdot \operatorname{Var}(\boldsymbol{g})}{\sigma^2} \\ &= \widehat{\beta}^2 \cdot \frac{(n-1) \cdot \operatorname{Var}(\boldsymbol{g})}{\operatorname{Var}(\boldsymbol{x}) - \widehat{\beta}^2 \cdot \operatorname{Var}(\boldsymbol{g})} \\ &= \widehat{\beta}^2 \cdot \frac{\operatorname{Var}(\boldsymbol{g})}{\operatorname{Var}(\boldsymbol{x})} \cdot \frac{(n-1)}{1 - \widehat{\beta}^2 \cdot \operatorname{Var}(\boldsymbol{g}) / \operatorname{Var}(\boldsymbol{x})} \end{aligned}$$

The standardised effect $\left(\widehat{\beta}_S\right)$ represents the effect on an SD/SD scale, i.e. expressing how many SD change in the phenotype one SD change of the genotype corresponds to. In other words, the squared standardised effect is the explained variance of the model. Thus,

$$\widehat{\beta}_S^2 = \widehat{\beta}^2 \cdot \frac{\operatorname{Var}(\boldsymbol{g})}{\operatorname{Var}(\boldsymbol{x})}$$

Substituting this effect into the formula for Z^2 above

$$Z^2 = \widehat{\beta}_S^2 \cdot \frac{n-1}{1 - \widehat{\beta}_S^2}$$

Inverting both sides gives

$$Z^{-2} = \frac{1}{n-1} \cdot \frac{1-\widehat{\beta}_S^2}{\widehat{\beta}_S^2} = \frac{1}{n-1} \cdot \left(\widehat{\beta}_S^{-2} - 1\right)$$

Thus,

$$(n-1) \cdot Z^{-2} + 1 = \widehat{\beta}_S^{-2}$$

which gives

$$\widehat{\beta}_S^2 = \frac{1}{(n-1) \cdot Z^{-2} + 1} = \frac{Z^2}{(n-1) + Z^2}$$

Since the test statistic for the standardised effect is the same as for the original-scale effect, therefore

$$Z^2 = \frac{\widehat{\beta}_S^2}{\operatorname{Var}\left(\widehat{\beta}_S\right)}$$

Thus,

$$\operatorname{Var}\left(\widehat{\beta}_S\right) = \frac{\widehat{\beta}_S^2}{Z^2} = \frac{1}{(n-1) + Z^2}$$

When n is large replacing $n-1$ with n in these formulae yields indistinguishable results, thus

$$\widehat{\beta}_S = \frac{Z}{\sqrt{n + Z^2}} \tag{1}$$

$$\operatorname{Var}\left(\widehat{\beta}_S\right) = \frac{1}{n + Z^2} \tag{2}$$

1. Swanson, S. A., Tiemeier, H., Ikram, M. A. & Hernán, M. A. Nature as a Trialist?: Deconstructing the Analogy Between Mendelian Randomization and Randomized Trials. *Epidemiology* **28**, 653 (2017).
2. Morris, T. T., Davies, N. M., Hemani, G. & Smith, G. D. Population phenomena inflate genetic associations of complex social traits. *Science Advances* **6**, eaay0328 (2020).
3. Zhernakova, D. V. *et al.* Identification of context-dependent expression quantitative trait loci in whole blood. *Nature Genetics* **49**, 139–145 (2017).
4. Leitsalu, L. *et al.* Cohort Profile: Estonian Biobank of the Estonian Genome Center, University of Tartu. *International Journal of Epidemiology* **44**, 1137–1147 (2015).
5. Dubois, P. C. A. *et al.* Multiple common variants for celiac disease influencing immune gene expression. *Nat Genet* **42**, 295–302 (2010).
6. Fehrmann, R. S. N. *et al.* Trans-eQTLs Reveal That Independent Genetic Variants Associated with a Complex Phenotype Converge on Intermediate Genes, with a Major Role for the HLA. *PLOS Genetics* **7**, e1002197 (2011).
7. Lonsdale, J. *et al.* The Genotype-Tissue Expression (GTEx) project. *Nat Genet* **45**, 580–585 (2013).
8. THE GTEX CONSORTIUM *et al.* The Genotype-Tissue Expression (GTEx) pilot analysis: Multitissue gene regulation in humans. *Science* **348**, 648–660 (2015).
9. THE GTEX CONSORTIUM. The GTEx Consortium atlas of genetic regulatory effects across human tissues. *Science* **369**, 1318–1330 (2020).
10. Tokolyi, A. *et al.* The contribution of genetic determinants of blood gene expression and splicing to molecular phenotypes and health outcomes. *Nat Genet* **57**, 616–625 (2025).

11. Holle, R., Happich, M., Löwel, H., Wichmann, H. E. & Group, null for the M. S. KORA - A Research Platform for Population Based Health Research. *Gesundheitswesen* **67**, 19–25 (2005).

12. Wichmann, H.-E., Gieger, C., Illig, T. & Group, null for the M. S. KORA-gen - Resource for Population Genetics, Controls and a Broad Spectrum of Disease Phenotypes. *Gesundheitswesen* **67**, 26–30 (2005).

13. Rathmann, W. *et al.* Incidence of Type 2 diabetes in the elderly German population and the effect of clinical and lifestyle risk factors: KORA S4/F4 cohort study. *Diabet Med* **26**, 1212–1219 (2009).

14. Schurmann, C. *et al.* Analyzing Illumina Gene Expression Microarray Data from Different Tissues: Methodological Aspects of Data Analysis in the MetaXpress Consortium. *PLOS ONE* **7**, e50938 (2012).

15. Raitakari, O. T. *et al.* Cohort profile: the cardiovascular risk in Young Finns Study. *Int J Epidemiol* **37**, 1220–1226 (2008).

16. Elovainio, M. *et al.* Childhood and adolescence risk factors and development of depressive symptoms: the 32-year prospective Young Finns follow-up study. *Journal of Epidemiology and Community Health (1979-)* **69**, 1109–1117 (2015).

17. Elovainio, M. *et al.* Activated immune–inflammatory pathways are associated with long-standing depressive symptoms: Evidence from gene-set enrichment analyses in the Young Finns Study. *Journal of Psychiatric Research* **71**, 120–125 (2015).

18. Ferrucci, L. *et al.* Subsystems contributing to the decline in ability to walk: bridging the gap between epidemiology and geriatric practice in the InCHIANTI study. *J Am Geriatr Soc* **48**, 1618–1625 (2000).

19. Ikram, M. A. *et al.* The Rotterdam Study. Design update and major findings between 2020 and 2024. *Eur J Epidemiol* **39**, 183–206 (2024).

20. Powell, J. E. *et al.* The Brisbane Systems Genetics Study: Genetical Genomics Meets Complex Trait Genetics. *PLOS ONE* **7**, e35430 (2012).

21. Powell, J. E. *et al.* Congruence of Additive and Non-Additive Effects on Gene Expression Estimated from Pedigree and SNP Data. *PLOS Genetics* **9**, e1003502 (2013).

22. Võsa, U. *et al.* Large-scale cis- and trans-eQTL analyses identify thousands of genetic loci and polygenic scores that regulate blood gene expression. *Nat Genet* **53**, 1300–1310 (2021).

23. University of Tartu. UT rocket. (2018) doi:10.23673/PH6N-0144.

24. Di Angelantonio, E. *et al.* Efficiency and safety of varying the frequency of whole blood donation (INTERVAL): a randomised trial of 45 000 donors. *Lancet* **390**, 2360–2371 (2017).

25. Moore, C. *et al.* The INTERVAL trial to determine whether intervals between blood donations can be safely and acceptably decreased to optimise blood supply: study protocol for a randomised controlled trial. *Trials* **15**, 363 (2014).

26. Fort, A. *et al.* MBV: a method to solve sample mislabeling and detect technical bias in large combined genotype and sequencing assay datasets. *Bioinformatics* **33**, 1895–1897 (2017).

27. Dobin, A. *et al.* STAR: ultrafast universal RNA-seq aligner. *Bioinformatics* **29**, 15–21 (2013).

28. Liao, Y., Smyth, G. K. & Shi, W. featureCounts: an efficient general purpose program for assigning sequence reads to genomic features. *Bioinformatics* **30**, 923–930 (2014).

29. Chang, C. C. *et al.* Second-generation PLINK: rising to the challenge of larger and richer datasets. *Gigascience* **4**, 7 (2015).

30. Astle, W. J. *et al.* The Allelic Landscape of Human Blood Cell Trait Variation and Links to Common Complex Disease. *Cell* **167**, 1415-1429.e19 (2016).

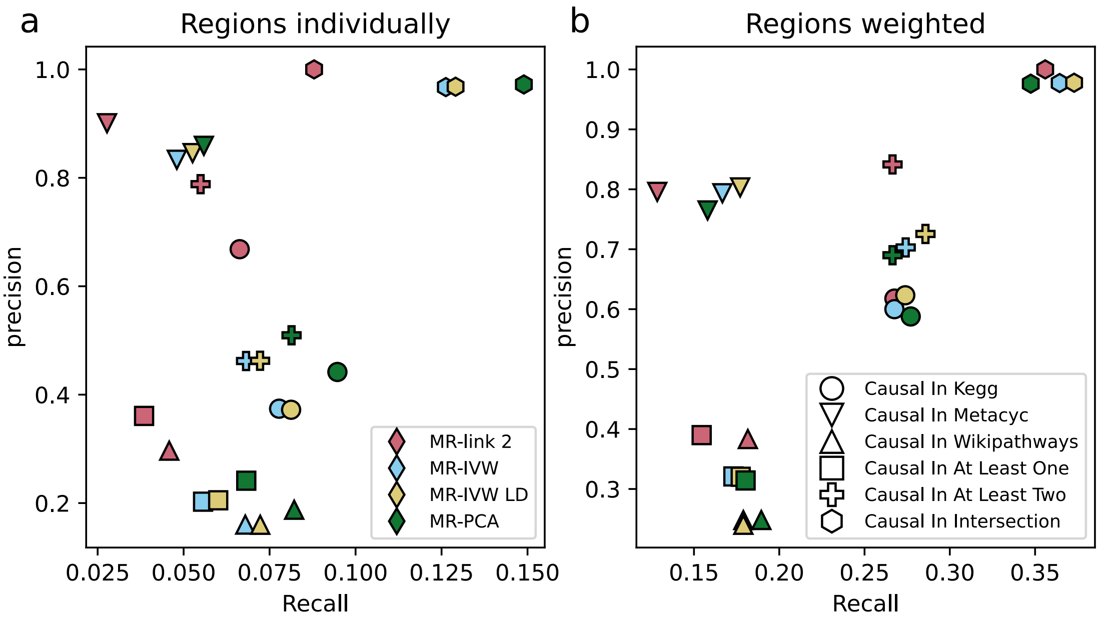

Supplementary Figure 1. Precision and recall for the *cis* MR methods in this study. Each point is colored based on the respective method and the shape of each point represents the ground truth the method is tested on. (**a**) The precision and recall at Bonferroni significance ($P < 2.3 \cdot 10^{-7}$) when considering each region individually (**Supplementary Data 6**). (**b**) The precision and recall at Bonferroni significance (P < $1.0 \cdot 10^{-6}$) when considering the inverse variance weighted estimate of each region together (**Supplementary Data 10**).

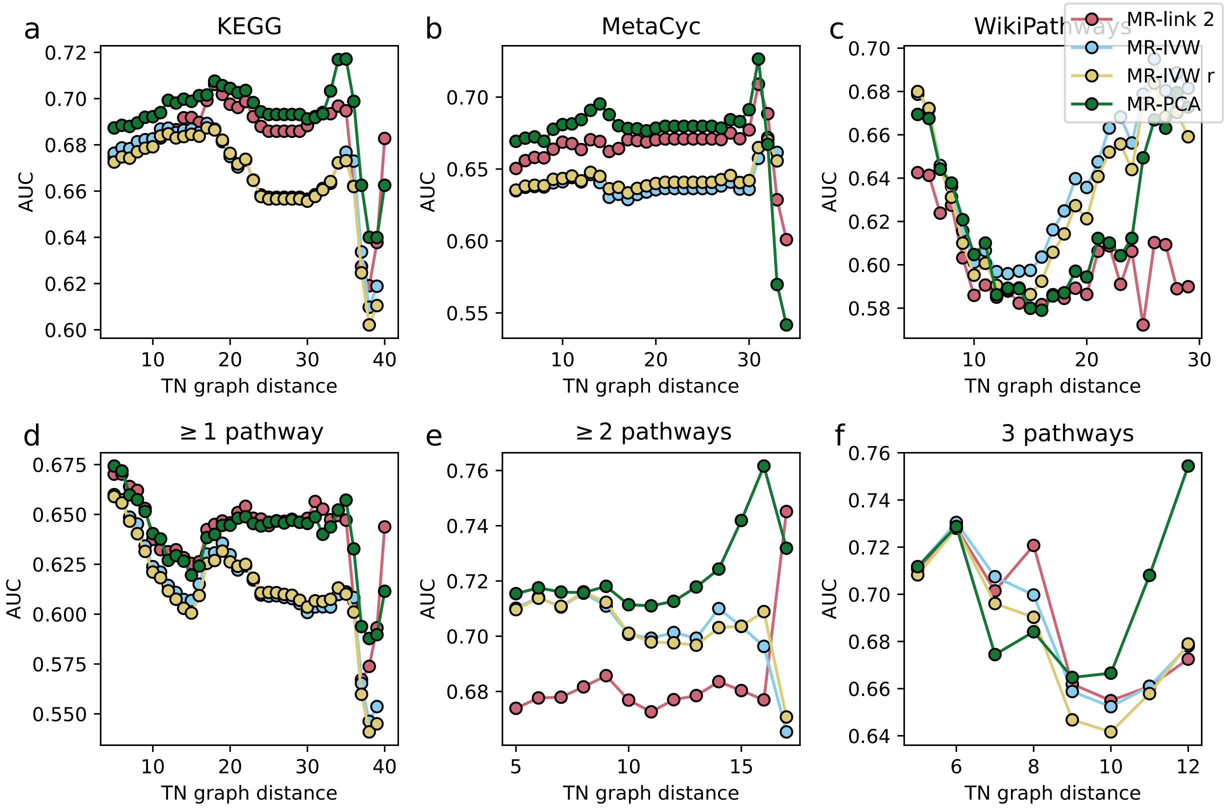

Supplementary Figure 2. The area under the receiver operator characteristic curve (AUC) of the *cis* MR methods in this study when meta-analyzed together. benchmarked against different databases (**a**-**c**) and database combinations (**d**-**f**). Only showing comparisons when there are more than 10 negatives per positive definition (**Supplementary Data 9**). (**a**) True causal links and false causal links from the KEGG pathway, (**b**) true causal links and false causal links from the MetaCyc pathway, (**c**) true causal links and false causal links from the WikiPathways pathway, (**d**) true causal links and false causal links that are present in any pathway definition, (**e**) true causal links and false causal links in at least two pathway definitions, (**f**) true causal links and false causal links that are shared in all pathways.

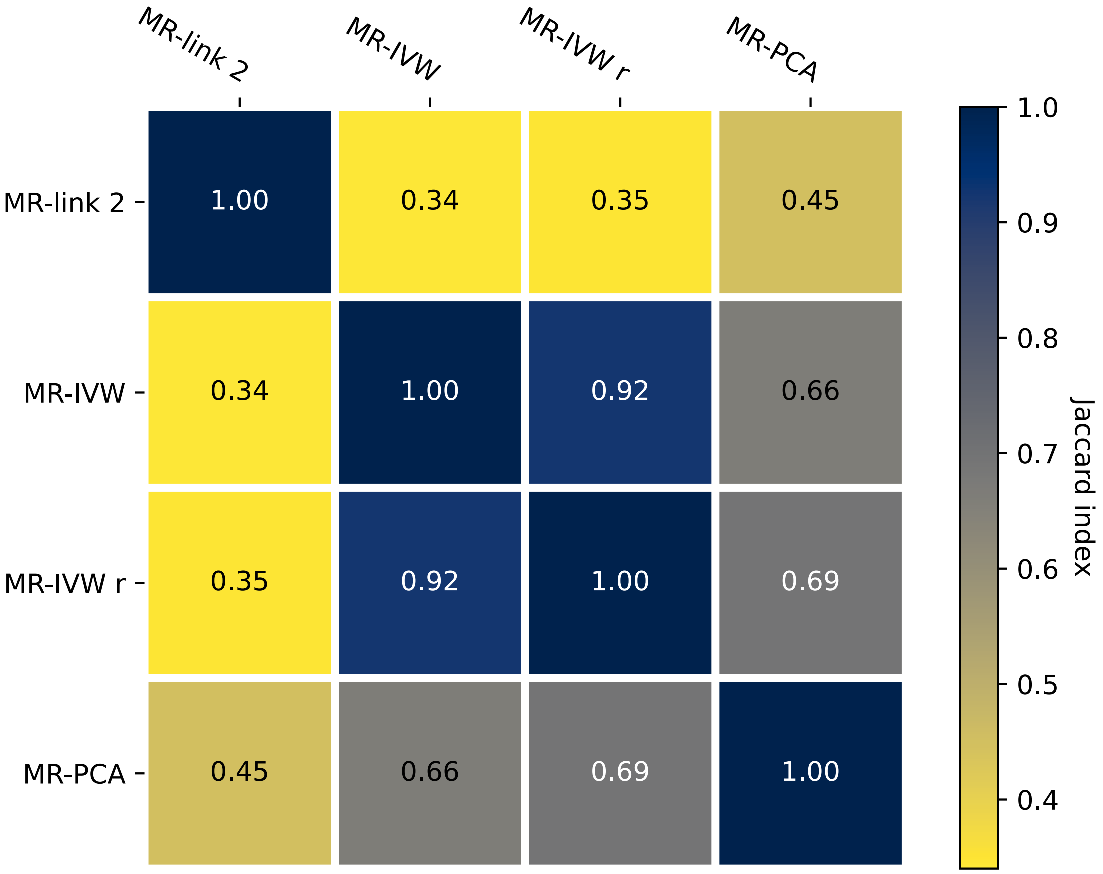

Supplementary Figure 3. The Jaccard matrix of the (weighted across regions) Bonferroni significant ($P < 1.0 \cdot 10^{-7}$) MR methods compared together. The Jaccard index is defined as sharing of the intersection of the set of causal relationships that two methods find divided by the union of the set of causal relationships (**Supplementary Data 10**).
