# Supplementary material for: MR-link-2: pleiotropy robust cis Mendelian randomization validated in three independent reference datasets of causality
Source: Nat Commun. 2025 Jul 3;16:6112. doi: 10.1038/s41467-025-60868-1 (PMC12229666; doi:10.1038/s41467-025-60868-1)
Supplement: Supplementary file 2 — Description of Additional Supplementary Files [file 41467_2025_60868_MOESM2_ESM.pdf]

Supplementary Data 1. Simulation results of MR-link-2. Detection rates for the two parameters MR-link-2 tests for ($\hat{\alpha}$ and $h^2_{\mathbf{Y}}$) and the number of successful simulation runs. Columns are as follow: "simulated-alpha": The α parameter simulated. "simulated-h^2_X": The simulated exposure heritability $h^2_{\mathbf{X}}$. "simulated-h^2_Y": The simulated outcome heritability $h^2_{\mathbf{Y}}$ that would violate the exclusion restriction. "simulated-n_ref": number of simulated individuals in the reference panel. When the number is 0 this means that the reference panel is simulated with full precision. "simulated-m_causal": The number of causal SNPs simulated for the exposure X and the outcome Y . "simulated-min(r_causal)": The minimum LD between causal SNPs. "simulated-max(r_causal)": The maximum LD between causal SNPs. "number_of_mr-link2_estimates": The number of successful estimates for MR-link-2. "MR-link_2_alpha_detection_rate": The detection rate for the causal estimate of MR-link-2 $\hat{\alpha}$ at P < 0.05. "MR-link_2_h^2_Y_detection_rate": The detection rate for MR-link-2 for the pleiotropy parameter $h^2_{\mathbf{Y}}$.

Supplementary Data 2. AUC comparisons in simulations of all the MR and coloc methods tested in this study. The columns are as follows: "simulated-alpha": The α parameter simulated. "simulated-h^2_X": The simulated exposure heritability $h^2_{\mathbf{X}}$. "simulated-h^2_Y": The simulated outcome heritability $h^2_{\mathbf{Y}}$ that would violate the exclusion restriction. "simulated-n_ref": number of simulated individuals in the reference panel. When the number is 0 this means that the reference panel is simulated with full precision. "simulated-m_causal": The number of causal SNPs simulated for the exposure $\mathbf{X}$ and the outcome $\mathbf{Y}$. "simulated-min(r_causal)": The minimum LD between causal SNPs. "simulated-max(r_causal)": The maximum LD between causal SNPs. "method": The method that makes the estimates. "number_of_null_estimates": The number of estimates when not simulating a causal effect. "number_of_non_null_estimates": The number of estimates when simulating a causal effect. "auc of the method": The area under the receiver operator characteristic curve for the methods estimates.

Supplementary Data 3. Regression of parameters of the simulation on the area under the receiver operator characteristic curve (AUC) of each method in this study. The columns are as follows: "explanatory": the explanatory variable, the parameter of the simulation, except when the variable is 'const', then it is the intercept, "Coef.": The coefficient of regression, "Std.Err.": The standard error of the coefficient of regression, "t": T statistic, "P>|t|": two-sided P value of the T statistic, "[0.025" and "0.975]" upper and lower confidence intervals of the coefficient, "method": The AUC of the method as the explained variable

Supplementary Data 4. Metabolites that are harmonized in this study, their identifiers in different databases, their metabolite quantitative trait locus (mQTL) study accession and indications where the metabolite has been used. The columns describe the harmonized name "harmonized_name", Identification in different pathway databases: ("hmdb_id", "inchikey" and "kegg_id"), the mQTL study accession "accession" and "study" and finally if and how the metabolite is used in analysis "used_as_outcome_in_metabolite_networks" indicates if the metabolite is found in the pathway databases and has a direct reaction with another metabolite. "used_as_exposure_in_metabolite_networks" indicates if the metabolite has an associated region from which it is possible to perform MR and finally "used_in_self_comparisons" is used to define if the metabolite is used in self-comparisons where an estimate is made based on the same metabolite measured in another mQTL study.

Supplementary Data 5. Regional metabolite self-comparisons. Here we report the causal estimates ("alpha_estimate", "se(alpha)") of different MR methods "method" and their P values from a two-sided Wald test: "P(alpha)" across comparisons of the same metabolites ('metabolite_name', "hmdb_id") measured in different studies ("exposure accession", "outcome_accession") across associated regions for the exposure ("region"). The column "included_in_bias_analysis" describes if the estimate is included in the bias analysis, as the P value is nominally significant ($P < 0.05$) and the metabolites are not considered in a reaction with one another considering the true positive dataset.

Supplementary Data 6. Regional metabolite self-comparisons. Here we report the causal estimates ("alpha_estimate", "se(alpha)") of different MR methods "method" and their P values from a two-sided Wald test: "P(alpha)" across comparisons of different metabolites for an exposure ("exposure_name", "exposure_hmdb_id", "exposure accession") and an outcome ("outcome_name", "outcome_hmdb_id", "outcome_accession") for each associated exposure region "associated region". The number of clumped variants in each region is denoted by "ivs in region".

Supplementary Data 7. Area under the receiver operator characteristic curves (AUCs) for each method's ("method") regional estimates evaluated independently. This is done for each pathway reference ("pwy") and minimum distance the metabolites are away from each other ("distance") in the extended graph. The AUC metric is performed on the respective number of "positives" and "negatives".

Supplementary Data 8. “precision” and “recall” for all the methods in both assessment of per locus independently and weighed together (“type”) across different pathway references “pwy”. These values are estimated for each individual method “method”.

Supplementary Data 9. Area under the receiver operator characteristic curves (AUCs) for each method (“method”) when regional estimates are weighted together. This is done for each pathway reference (“pwy”) and minimum distance the metabolites are away from each other (“distance”). The AUC metric is performed on a number of “positives” and “negatives”.

Supplementary Data 10. Metabolite to metabolite estimates, all regions of an exposure outcome combination weighted together. Here we report the causal estimates (“alpha_estimates”, “se(alpha)”of different MR methods “method” and their P values from an inverse variance weighted fixed effect meta-analysis: “P(alpha)” across comparisons of different metabolites for an exposure (“exposure_name”, “exposure_hmdb_id”, “exposure accession”) and an outcome (“outcome_name”, “outcome_hmdb_id”, “outcome_accession”). The causal relationship as defined by our true positive reference (“KEGG direct reaction”,, “MetaCyc direct reaction”, and “WikiPathways direct reaction”) The number of regions weighted together is represented by “# Meta-analyzed regions”.

Supplementary Data 11. The causal estimates all complex-complex trait combinations. Here we report the causal estimates (“Weighted alpha estimates”, “se(alpha)”) of different MR methods “Method” and their P values from a two-sided Wald test: “P(alpha)” across comparisons of different metabolites for an exposure (“exposure_name”) and an outcome (“outcome_name”), and if the combination is considered causal by Morrison et al.[9] (“Combination considered causal”, “Combination considered non causal”).

Supplementary Data 12. Detection rates at two-sided Wald test P < 0.05 per associated region (“Ratio of regions P < 0.05’) of a *cis* MR estimate for all complex trait combinations (“Exposure trait”, “Outcome trait”) that are considered causal or not causal (“Considered causal”), for each *cis* MR method tested “method”.

Supplementary Data 13. Spearman correlation ("Spearman Correlation", "p value") between the pleiotropic estimates ($\hat{h}_{\mathbf{Y}}$, where $\hat{h}_{\mathbf{Y}}$ P < 0.05) compared to each *cis* methods' ("method") absolute regional (α_r) estimate deviation from meta-analyzed ($\bar{\alpha}$) across all comparisons ("number of comparisons")

Supplementary Data 14. *cis* Mendelian randomization (MR) and coloc results of 6 examples of true positives and true negative loci. The Associated region from which the MR and coloc region is derived ("associated region"), "exposure", "outcome" and relevant result for each individual method ("p(MR-link-2)", "p(MR-IVW) ", "p(MR-IVW LD) ", "p(MR-PCA) ", "coloc PP.H4.abf", "coloc susie max(PP.H4.abf)"). All P values are from a two-sided Wald test.

Supplementary Data 15. Inverse variance weighted (across associated regions) analysis of cell types on gene expression and vice versa. Here we report the causal estimates ("Weighted alpha estimates", "se(alpha)") of different MR methods "Method" and their P values from an inverse variance weighted fixed effect meta-analysis: "P(alpha)" across comparisons of different metabolites for an exposure ("exposure_name",) an outcome ("outcome_name") and if the causal combination is considered "causal". We consider the comparison causal if cell type composition differences influence their markergene expression.

Supplementary Data 16. The summary statistics of all the complex traits that are used in this study, including the trait of interest "Complex trait", the origin of the summary statistics file "Publication", the maximum sample size for the trait and the doi identifier of the publication.

Supplementary Data 17. Number of individuals stratified by their sex per cohort in the eQTLGen Consortium data used in this study.

Supplementary Data 18. Cell type ("Celltype") and marker gene ("markergene") reference adapted from the Azimuth reference. These cell type and markergene combinations are considered the true causal links that are used in the cell type analysis.
