# Supplementary material for: MR-link-2: pleiotropy robust cis Mendelian randomization validated in three independent reference datasets of causality
Source: Nat Commun. 2025 Jul 3;16:6112. doi: 10.1038/s41467-025-60868-1 (PMC12229666; doi:10.1038/s41467-025-60868-1)
Supplement: Supplementary file 21 — Reporting Summary [file 41467_2025_60868_MOESM21_ESM.pdf]

nature portfolio

Corresponding author(s): Zoltán Kutalik

Last updated by author(s): 28 March 2025

Reporting Summary

Nature Portfolio wishes to improve the reproducibility of the work that we publish. This form provides structure for consistency and transparency in reporting. For further information on Nature Portfolio policies, see our Editorial Policies and the Editorial Policy Checklist.

Statistics

For all statistical analyses, confirm that the following items are present in the figure legend, table legend, main text, or Methods section.

n/a	Confirmed	
☐	☒	The exact sample size (*n*) for each experimental group/condition, given as a discrete number and unit of measurement
☒	☐	A statement on whether measurements were taken from distinct samples or whether the same sample was measured repeatedly
☐	☒	The statistical test(s) used AND whether they are one- or two-sided<br>*Only common tests should be described solely by name; describe more complex techniques in the Methods section.*
☐	☒	A description of all covariates tested
☐	☒	A description of any assumptions or corrections, such as tests of normality and adjustment for multiple comparisons
☐	☒	A full description of the statistical parameters including central tendency (e.g. means) or other basic estimates (e.g. regression coefficient) AND variation (e.g. standard deviation) or associated estimates of uncertainty (e.g. confidence intervals)
☐	☒	For null hypothesis testing, the test statistic (e.g. *F*, *t*, *r*) with confidence intervals, effect sizes, degrees of freedom and *P* value noted<br>*Give P values as exact values whenever suitable.*
☒	☐	For Bayesian analysis, information on the choice of priors and Markov chain Monte Carlo settings
☒	☐	For hierarchical and complex designs, identification of the appropriate level for tests and full reporting of outcomes
☐	☒	Estimates of effect sizes (e.g. Cohen's *d*, Pearson's *r*), indicating how they were calculated

Our web collection on statistics for biologists contains articles on many of the points above.

Software and code

Policy information about availability of computer code

Data collection	Primary data collection was done using public resources, using data downloaded from the internet.<br>Harmonization of this data was done using custom python scripts (version 3.11) and plink v1.90b6.26<br><br>The eQTLGen Consortium provided data for this study using data through their custom pipelines (See Supplementary Notes and https://eqtlgen.github.io/eqtlgen-web-site/eQTLGen-p2-cookbook.html)<br><br>The genotype information for LD calculation was based on the UK10K data resource, which was downloaded from accession numbers: EGAD00001000740, EGAD00001000741
Data analysis	We analyzed data using our newly presented MR-link-2 methodology which has been implemented into a custom python 3.11 script. The code for which can be found at: https://github.com/adriaan-vd-graaf/mrlink2. We have also used PLINK vl.90b6.26

For manuscripts utilizing custom algorithms or software that are central to the research but not yet described in published literature, software must be made available to editors and reviewers. We strongly encourage code deposition in a community repository (e.g. GitHub). See the Nature Portfolio guidelines for submitting code & software for further information.

Data

Policy information about availability of data

All manuscripts must include a data availability statement. This statement should provide the following information, where applicable:

- Accession codes, unique identifiers, or web links for publicly available datasets
- A description of any restrictions on data availability
- For clinical datasets or third party data, please ensure that the statement adheres to our policy

The code for simulations and working examples for MR-link-2 and the other cis- causal inference methods are available at: https://github.com/adriaan-vd-graaf/ 1 mrlink2. The summary statistics for the metabolite analysis, the complex trait analysis and the blood cell type composition phenotypes are available from the respective source publications (Supplementary Table 15). The data availability of the each of the eQTLGen Consortium cohorts is listed in the (Supplementary Note). The genotype information underlying the LD matrices for the UKl0K data resource were downloaded from (EGAD00001000740, EGAD00001000741).

Research involving human participants, their data, or biological material

Policy information about studies with human participants or human data. See also policy information about sex, gender (identity/presentation), and sexual orientation and race, ethnicity and racism.

Reporting on sex and gender	The conclusions of this study are not specific to any sex and or gender.<br>Here, we only analyze autosomal genetics. This is the genetic information that is not specific to sex and or gender.<br>Publicly available summary statistics state their sex and or gender covariate adjustment in their base publication.<br>The eQTLGen Consortium performs a covariate adjustment based on the individuals genetically derived sex. If the individuals genetic sex does not match the reported sex, the individual is removed from analysis.<br>(https://eqtlgen.github.io/eqtlgen-web-site/eQTLGen-p2-cookbook.html)<br>The individual numbers and their sex per cohort of the eQTLGen Consortium can be found in Supplementary Data 17
Reporting on race, ethnicity, or other socially relevant groupings	For the publicly available information, we have analyzed individuals of European genetic ancestry. How this was defined can can be found in the relevant base publications (Supplementary Table 16). This choice has been made to ensure that the linkage disequilibrium reference matched the publicly available summary statistics<br>The eQTLgen consortium inclusion has not been selected for ancestry.
Population characteristics	We did not recruit any participants for this study, and therefore refer to the relevant base publications for their population characteristics. Details can be found in the base publications of the studies (Supplementary Data 16).<br>The eQTLGen Consortium population characteristics can be found in their base publication and the Supplementary Note.
Recruitment	We did not recruit any participants for this study, and therefore refer to the relevant base publications for their population characteristics. Details can be found in the base publications of the studies (Supplementary Data 16).<br>The eQTLGen Consortium population characteristics can be found in their base publication and the Supplementary Note.
Ethics oversight	We used publicly available information for which the ethical oversight committees are referenced in their base publications (Supplementary Table 15).<br>The ethical oversight approvals for the eQTLgen consortium are listed below:<br>The eQTLGen phase II research activities involving Estonian Biobank participant data (two EstBB cohorts) have been carried out under the ethical approval nr. 1.1-12/655 and its extension 1.1- 12/490 by the Estonian Committee on Bioethics and Hu man Research (Estonian Ministry of Social<br>1330 Affairs), using data according to release application number 554 from the Estonian Biobank.

Note that full information on the approval of the study protocol must also be provided in the manuscript.

Field-specific reporting

Please select the one below that is the best fit for your research. If you are not sure, read the appropriate sections before making your selection.

[x] Life sciences [ ] Behavioural & social sciences [ ] Ecological, evolutionary & environmental sciences

For a reference copy of the document with all sections, see nature.com/documents/nr-reporting-summary-flat.pdf

Life sciences study design

All studies must disclose on these points even when the disclosure is negative.

Sample size	We have used summary statistics from multiple cohorts. These files generally contain the number of observations per marker for each genetic variant. We have used this as the sample size that is used later for our likelihood function. When this information was not avaiable, we set this information to the maximum sample size as reported by the base publication.<br><br>The freeze III of the eQTLgen consortium contains a total of 14,855 individuals derived from 19 cohorts of gene expression.

Data exclusions	We have excluded summary statistic files when they did not match the genetic ancestry of the linkage equilibrium reference. Furthermore, we have excluded genetic variants when they did not conform to the following inclusion criteria: No allelic match to our linkage disequilibrium reference, minor allele frequency > 0.5% and the association must be derived from more than 95% of the maximum number of individuals for the phenotype.<br>The eQTLgen Consortium removes genetic variants based on the following criteria (https://eqtlgen.github.io/eqtlgen-web-site/eQTLGen-p2-cookbook.html): call-rate>0.95, Hardy-Weinberg P>le-6, MAF>0.01<br>Furthermore, eQTLgen Consortium individuals are excluded from analysis when they match the following criteria:<br>- genetic missingness > 0.05<br>- unclear genetic sex,<br>- excess heterozygosity (+/- 3 standard deviations from the mean)<br>- one individual is kept when a pair is found that is related more than a 3rd degree relative.<br>- individuals that are genetic outliers within the subcohort.<br>- individuals whose genetic sex does not match their reported sex.
Replication	We have not performed post-hoc replication of findings.<br>Whenever multiple observations of the same trait were available, we performed the analysis independently on all traits and report all estimates.
Randomization	We did not perform randomization of research subjects. Our analysis is based on observational studies.
Blinding	As our analysis has been based on observational data, we have not performed blinding

Reporting for specific materials, systems and methods

We require information from authors about some types of materials, experimental systems and methods used in many studies. Here, indicate whether each material, system or method listed is relevant to your study. If you are not sure if a list item applies to your research, read the appropriate section before selecting a response.

Materials & experimental systems

n/a	Involved in the study
☒	☐ Antibodies
☒	☐ Eukaryotic cell lines
☒	☐ Palaeontology and archaeology
☒	☐ Animals and other organisms
☒	☐ Clinical data
☒	☐ Dual use research of concern
☒	☐ Plants

Methods

n/a	Involved in the study
☒	☐ ChIP-seq
☒	☐ Flow cytometry
☒	☐ MRI-based neuroimaging

Plants

Seed stocks	n/a
Novel plant genotypes	n/a
Authentication	n/a
